# Variation among populations in the immune protein composition of mother’s milk reflects subsistence pattern

**DOI:** 10.1093/emph/eoy031

**Published:** 2018-10-13

**Authors:** Laura D Klein, Jincui Huang, Elizabeth A Quinn, Melanie A Martin, Alicia A Breakey, Michael Gurven, Hillard Kaplan, Claudia Valeggia, Grazyna Jasienska, Brooke Scelza, Carlito B Lebrilla, Katie Hinde

**Affiliations:** 1 Department of Human Evolutionary Biology, Harvard University, 11 Divinity Avenue, Cambridge, MA, USA; 2 Department of Anthropology, University of Illinois at Chicago, 1007 West Harrison Street, Chicago IL, USA; 3 Chemistry Department, University of California Davis, 2465 Chemistry Annex, One Shields Avenue, Davis, CA, USA; 4 Department of Anthropology, Washington University in St Louis, Campus Box 1114, One Brookings Drive, St Louis, MO, USA; 5 Department of Anthropology, University of California Santa Barbara, Santa Barbara, CA, USA; 6 Department of Anthropology, University of Washington, 314 Denny Hall, Box 353100, Seattle, WA, USA; 7 Department of Anthropology, University of New Mexico, MSC01-1040, 1 University of New Mexico, Albuquerque, NM, USA; 8 Department of Anthropology, Yale University, 10 Sachem Street, New Haven, CT, USA; 9 Department of Environmental Health, Faculty of Health Sciences, Jagiellonian University Medical College, ul. Grzegorzecka 20, Krakow, Poland; 10 Department of Anthropology, University of California Los Angeles, 341 Haines Hall, Box 951553, Los Angeles, CA, USA; 11 School of Human Evolution and Social Change; 12 Center for Evolution and Medicine, Arizona State University, Tempe, AZ, USA

**Keywords:** milk immunofactors, human milk, maternal ecology, SIgA, lactoferin

## Abstract

**Lay Summary:**

Adaptive immune proteins in mothers’ milk are more variable than innate immune proteins across populations and subsistence strategies. These results suggest that the immune defenses in milk are shaped by a mother’s environment throughout her life.

**Background and objectives:**

Mother’s milk contains immune proteins that play critical roles in protecting the infant from infection and priming the infant’s developing immune system during early life. The composition of these molecules in milk, particularly the acquired immune proteins, is thought to reflect a mother’s immunological exposures throughout her life. In this study, we examine the composition of innate and acquired immune proteins in milk across seven populations with diverse disease and cultural ecologies.

**Methodology:**

Milk samples (*n* = 164) were collected in Argentina, Bolivia, Nepal, Namibia, Philippines, Poland and the USA. Populations were classified as having one of four subsistence patterns: urban-industrialism, rural-shop, horticulturalist-forager or agro-pastoralism. Milk innate (lactalbumin, lactoferrin and lysozyme) and acquired (Secretory IgA, IgG and IgM) protein concentrations were determined using triple-quadrupole mass spectrometry.

**Results:**

Both innate and acquired immune protein composition in milk varied among populations, though the acquired immune protein composition of milk differed more among populations. Populations living in closer geographic proximity or having similar subsistence strategies (e.g. agro-pastoralists from Nepal and Namibia) had more similar milk immune protein compositions. Agro-pastoralists had different milk innate immune protein composition from horticulturalist-foragers and urban-industrialists. Acquired immune protein composition differed among all subsistence strategies except horticulturist-foragers and rural-shop.

**Conclusions and implications:**

Our results reveal fundamental variation in milk composition that has not been previously explored in human milk research. Further study is needed to understand what specific aspects of the local environment influence milk composition and the effects this variation may have on infant health outcomes.

## BACKGROUND AND OBJECTIVES

Born with immature immune systems, human neonates are vulnerable to infection, particularly infections caused by respiratory and gastrointestinal pathogens. Breastfed infants, however, have fewer and less severe illnesses than do formula-fed infants [1, 2]. Mother’s milk contains immunologically active molecules that play critical roles in protecting the infant and educating the infant’s naïve immune system. Many of these components are active in mucosal defense against infection, including preventing proliferation of pathogens and promoting maturation of the mucosal barrier [3]. Although these molecules can resist digestion and retain biological activity in the infant’s digestive tract (e.g. lactoferrin, Secretory IgA [SIgA]) (reviewed in [4, 5]), digestion is necessary for the activation of other immunologically protective compounds (e.g. lactalbumin) [6]. Milk bioactives may also indirectly shape the development of the infant’s immune function through interactions with the infant’s gut microbiota [7]. Additionally, these immunologically active milk molecules seemingly shape the maturation and regulation of the infant’s gut immunity, which is important for the regulation of the immune system overall [8]. Human milk modulates the immune response of intestinal epithelial cells to microbial components *in vitro*, though the direction of this modulation varies with the mother’s country of origin [9]. The immunological protection provided by human milk is likely shaped by a mother’s current and past environment to promote the infant’s survival during the earliest encounters in a complex microbial and pathogenic postnatal landscape. In this study, we examine the composition of milk immune proteins across diverse populations and subsistence patterns to explore how the immune protection in mother’s milk may be shaped by the maternal environment.

The immune protection provided by mother’s milk consists of two layers of defense, the innate immune system and the acquired (or adaptive) immune system. The innate immune system is evolutionarily ancient and provides the first line of defense against pathogens [10]. These broad, nonspecific defenses have been shaped by selection to respond to highly conserved features of pathogens, and this response does not change with repeated exposure. Among the most important innate immune proteins in milk are lactoferrin, lysozyme and lactalbumin (Table 1). The acquired immune system is characterized by specificity and memory, producing specific defenses to pathogens that an individual has been exposed to previously in their lifetime [17, 18]. Though these acquired defenses initially require more time to respond, immunological memory allows for stronger and more efficient immune responses upon repeated exposures to a previously encountered pathogen. Thus, acquired immune proteins reflect both an individual’s history of immunological exposure and their acute immune activation. Immunoglobulins are the most well-known acquired immune proteins in milk (Table 1). Antibodies in milk are largely specific for pathogens encountered by maternal mucosal tissues, making them reflective of the environment that an individual has encountered throughout her life [19, 20]. Milk immunoglobulins provide critical passive immunity to the infant after birth, but may also prime the infant’s own developing immune system [21]. In this way, milk functions as an intergenerational bridge that carries acquired immunity to the infant from the mother’s lifetime of exposure. This bridge may be especially important in the human lineage, as proteins that are associated with gastrointestinal, brain and immune development are more abundant in human milk than macaque milk [22]. Athough data from more closely related primate species are needed, Beck and colleagues propose that this is among the first evidence that disease and nutritional ecology may contribute to derived features of human milk proteins [22].

**Table 1.** Selected functions of acquired and innate immune proteins in human milk

Protein	Functions	References
*Innate Immune Proteins*		
Lactoferrin	Bacteriostatic, antibacterial, and antiviral activities Modulates inflammation Regulates intestinal cell proliferation and differentiation	[11, 12]
Lysozyme	Disrupts cell membranes in gram-positive bacteria Acts in conjunction with lactoferrin to kill gram-negative bacteria Stimulates maturation of intestinal tract Promotes beneficial gut microbiome profiles in animal models	[11, 13, 14]
Lactalbumin	Primary role as a regulatory subunit of lactase synthase Alpha-lactalbumin can also combine with oleic acid to form a protein complex (HAMLET) capable of killing tumor cells	[6, 15]
*Acquired Immune Proteins*		
SIgA	Primary antibody for mucosal defense and the most abundant immunoglobulin in human milk Specific antibodies have been identified to most major classes of pathogens, including bacteria, viruses, fungi, and yeasts Reflect pathogens encountered by the mother during her lifetime Thought to have lasting beneficial effects on the infants’ gut microbiome and immune system regulation	[3, 16]
IgG	Present in human milk at much lower concentrations than SIgA Unlikely to resist digestion in the small intestine and likely plays a small role in providing passive immunity to the infant	[11]
IgM	Present in human milk at much lower concentrations than SIgA Unlikely to resist digestion in the small intestine and likely plays a small role in providing passive immunity to the infant	[11]

The pathogens that humans encounter are determined by our physical and cultural environments. Human pathogen species diversity decreases with increasing distance from the equator, similar to the latitudinal species gradients identified for many other taxonomic groups [23]. Climatic factors, such as temperature and precipitation, are hypothesized to drive this distribution by influencing the pathogens endemic to an area, the seasonality of outbreaks, and/or the presence of vectors or alternative hosts [23–26]. As a result, people living in tropical areas with higher temperatures and precipitation are likely to be exposed to a greater diversity of pathogens than people in more temperate areas [23]. Human behavior, including cultural practices, also influences pathogen exposure [27, 28]. Throughout our species’ evolutionary history, cultural practices have engineered new niches and affected the global distribution of species, including of pathogen species (reviewed in [29]). Thus, considering the cultural ecology of a population is likely to be an important part of understanding mothers’ local environments.

In industrialized, wealthy countries, public health initiatives (i.e. health care, potable water, sewage containment and waste removal) reduce pathogen exposures and contribute to lower rates of infectious diseases and parasitic infections, including those that co-evolved with humans to modulate immune response [30]. Lack of exposure to these microbial ‘old friends’, particularly in urban areas, is associated with relatively higher rates of allergies and immunoregulatory disorders [30]. Populations with non-industrialized subsistence strategies tend to have higher pathogen exposure and burdens, though the sources and types of pathogens, and their effects on the immune system, are variable. Subsistence agriculturalists encounter pathogens through soil cultivation and the use of human and domesticated animal feces for fertilizer [28]. Agriculture also supports larger and more densely populated communities, which permit crowd diseases (e.g. measles, tuberculosis) that are not common among other subsistence patterns and activate pro-inflammatory, Th1-type (intracellular pathogen-directed) immune responses [30, 31]. Pastoralists are exposed to zoonotic diseases through routine close contact with domestic animals as well as exposures to animal blood or secretions during butchering and the consumption of meat and raw milk [32, 33]. Hunter-gatherers and other groups that consume wild meat can also be exposed to zoonotic diseases from contact with wild animals, especially through injuries (e.g. scratches, bites) acquired during hunting and exposures during butchering [34]. Among hunter-gatherers and horticulturalist-foragers, parasitic (including helminth) and fungal infections are common. When compared with western populations, some of these groups have genetic profiles that favor Th2-type (extracellular pathogen-directed) immune responses and elevated levels of immune molecules throughout life, particularly those involved in response to extra-cellular parasite infections [35, 36]. Subsistence pattern has also been correlated with gut microbiome composition among closely related, rural African populations [37], as well as populations that share a subsistence patterns but are located on different continents [38]. As the gut microbiome plays a considerable role in shaping immune function, differences in microbiome could translate to differences in immune function among populations. Due to these broadly documented phenomena, human biologists and anthropologists use subsistence pattern as a rough proxy for the pathogen pressure that individuals within a population may encounter, especially when national health statistics are unlikely to reflect the experiences of traditionally living populations [39]. It is worthwhile to remember, however, that pathogen exposures are not exclusive to one subsistence strategy and many populations engage in several types of subsistence activities. For example, agro-pastoralists and subsistence agriculturalists can both be exposed to zoonotic diseases from domesticated animals as well as to pathogens during soil cultivation though the patterns of exposure are still likely to differ across these subsistence strategies based on factors such as the number of animals present and proportion of time spent in each activity.

Public health initiatives also examine environmental characteristics to determine the effects of environment on human health. The environmental characteristics of interest to health researchers are proxies of the same kinds of phenomena that anthropologists evaluate through a theoretical lens. Biomarkers of immune function, or more frequently morbidity/disease rates, are correlated with characteristics of the broader environment (e.g. high income countries vs. the global south) or the household environment (e.g. the presence or absence of dirt floors or toilets) [40]. These parameters often directly or indirectly relate to differences in pathogen exposure and public health interventions aim to reduce morbidity or mortality by reducing pathogen exposures [40, 41]. Previous studies have investigated how human milk immunofactor concentrations are influenced by environmental characteristics. Milk immunofactor concentrations have been found to vary with maternal country of residence [42–45] or maternal country of origin [20, 46, 47, but see 9]. Most of these studies, however, have investigated or compared urban areas of western, industrialized countries. Thus, our understanding of how maternal environment shapes variation in the immunofactors in milk is limited.

In this study, we compared the composition of select immune proteins in human milk across populations and subsistence strategies to characterize how immune proteins provided via mother’s milk vary across maternal environments. To do this, we analyzed the levels of innate (lactalbumin, lysozyme and lactoferrin) and acquired (SIgA, IgG and IgM) immune proteins in human milk across seven populations that reflect diverse cultural and disease ecologies. Importantly, all samples were analyzed in the same laboratory at the same time, allowing us to make direct comparisons among populations. Due to the differences in the origins and functions of innate and acquired immunity, we predicted that acquired immune protein composition would differ more across populations and subsistence patterns than innate immune protein composition in milk across populations living in different environments. Characterizing how immune protein concentrations in milk vary across populations and environments is the first step in understanding how specific aspects of the maternal environment relate to the immune protection provided by mothers’ milk. To the best of our knowledge, this is the first study to look at human milk immune protein concentrations in relationship to subsistence strategy.

## METHODOLOGY

### Populations

Milk samples were collected from mother–infant dyads among populations in the USA, Argentina, Philippines, Poland, Bolivia, Namibia and Nepal between 2007 and 2013 (Supplementary Material S1). The lifestyle and environment of these populations vary with respect to their participation in agriculture, contact with wild and domesticated animals, and access to sanitary infrastructure and medical care (Table 2) with greater ethnographic detail provided in the Supplementary Material S1. Mother–infant dyads varied in a number of demographic parameters (Table 3).

**Table 2.** Variation in pathogen exposures across populations

	USA	Argentina	Philippines	Poland	Bolivia	Namibia	Nepal
	Boston	Qom	Cebu	Rural Polish	Tsimane	Himba	Nubri Tibetan
Routine exposure to livestock			**✓**	**✓**		**✓**	**✓**
Subsistence Horticulture/Agriculture			**✓**	**✓**	**✓**	**✓**	**✓**
Routine hunting of wild animals					**✓**		
Dirt flooring in the home		**✓**			**✓**	**✓**	
Lack of indoor plumbing		**✓**	**✓**		**✓**	**✓**	**✓**
Limited access to modern, western biomedical care					**✓**	**✓**	**✓**

This table is a visual summary of environmental characteristics that are suspected to contribute to increased pathogen exposure within these populations. No mark indicates the exposure is not present or rare in the population, a gray check mark (**✓**) indicates the exposure is variably present in the population, and a black check mark (**✓**) indicates the exposure is widespread in the population.

**Table 3.** Summary of participant characteristics

Country	Population	*n*	Infant sex (male) *n* (%)	Infant age (days) (*M* ± *SEM*)	Parity (*M* ± *SEM*)	Primiparous *n* (%)	Subsistence Strategy	References
USA	Boston, MA	21	7 (33%)	197.8 ± 21.5	1.7 ± 0.2	11 (52%)	Urban-industrial	
Argentina	Qom (Toba)^a^	18	9 (50%)	236.5 ± 22.8	3.7 ± 0.5	3 (17%)	Rural-shop	[5, 48]
Philippines	Cebu	17	8 (47%)	190.0 ± 25.2	2.0 ± 0.2	4 (24%)	Rural-shop	[49]
Poland	Mogielica Human Ecology Study Site	22	12 (55%)	187.4 ± 20.3	2.2 ± 0.3	10 (45%)	Rural-shop	[50, 51]
Bolivia	Tsimane^a^	47	28 (60%)	265.1 ± 23.4	4.2 ± 0.4	6 (13%)	Horticulturalist- forager	[36]
Namibia	Himba^a^	11	5 (45%)	172.0 ± 40.7	3.8 ± 0.9	2 (18%)	Agro-pastoralist	[52, 53]
Nepal	Nubri Tibetan^a^	28	13 (46%)	240.5 ± 34.0	2.1 ± 0.3	13 (46%)	Agro-pastoralist	[54, 55]
TOTAL		164	82 (50%)	224.7 ± 10.8	2.9 ± 0.2	49 (21%)		

a Indicates an indigenous population.

### Subsistence strategy assignment

Populations were binned into one of four subsistence patterns: urban-industrialists, rural-shop, agro-pastoralists or horticultural-forager (Table 3). Anthropological studies of nutritional ecology have prioritized investigating traditional subsistence activities, but most traditional societies now participate in mixed economies to some extent [56]. Increased urbanization and engagement in wage labor is associated in many parts of the world with nutritional transitions towards a more westernized diet [57]. The category of ‘rural-shop’ encompasses populations that purchase much of their food from small local groceries that have limited inventory, and are not directly comparable to an urban population in a westernized, industrialized nation. For example, the Qom (formerly Toba) people of Argentina were traditionally hunter-gatherers, but now live in government designated territories. Though foraging is still practiced in some rural territories, in peri-urban barrios, most food is purchased with governmental aid and supplemental income from men’s paid labor and women’s sales of artisanal crafts [58]. Diets tend to be monotonous and include calorie-dense food, such as fried dough, noodles and potatoes [48, 59]. The Mogielica Human Ecology Study Site in Poland is located in a region historically characterized by peasant subsistence farming. Despite rapidly increasing participation in wage labor, 65% of household surveyed in 2009–10 reported growing at least some of their own produce [51]. Small shops in the area provide limited selections of fresh produce, meat and dairy products, and larger selections of canned and dry goods, sweets, juices, soft drinks and alcohol. Participants in the Cebu Longitudinal Health and Nutrition Survey (Cebu Study, Philippines) were drawn from the greater metropolitan area of Cebu, Philippines. Approximately half the participants were from urban areas engaged in wage labor, and half from surrounding agrarian communities practicing small scale farming. Both groups had access to a variety of commercially produced foods as well as local produce, eggs and fish.

### Participant characteristics

Women included in this study were nursing children from a singleton pregnancy and reported no indications of mastitis at the time of milk collection. Mothers of infants under 2 weeks of post-natal age or over 2 years of post-natal age were excluded from the study. Among the Himba, Nubri Tibetans, Tsimane and Qom, ‘on-demand’ breastfeeding for 2–3 years is typical and commercial infant formulas are rarely available, though complementary foods are usually introduced when the infant is between four and six months old [48, 55, 60, 61]. Breastfeeding duration or initiation rates can be much more variable within the other populations. Although nearly all Filipino women in Cebu initiate breastfeeding and many continue to breastfeed for at least 18 months, the median duration is around 12 months [62, 63]. Similarly, Polish women living in the Mogielica Human Ecology Study Site area typically breastfeed for around 12 months ([64]; unpublished data). However, within the United States, 79% of mothers ever breastfeed and only 27% continue for at least 12 months. Factors such as mothers’ socioeconomic status, race/ethnicity, level of education, geographic location, and family and social support are well-known to contribute to considerable variation in breastfeeding rates and duration in the USA [65].

### Milk collection

Milk samples (*n* = 166) were collected using a standardized procedure. To control for diurnal changes in milk composition, all samples were collected in the morning. Mothers were asked to nurse their infants from the sample breast 2 h prior to the sample collection and only nurse from the non-sample breast to allow for a standardized pooling period for milk in the breast. Participants provided mid-feed milk samples following Neville and colleagues [66]. Briefly, infants suckled from the sample breast for 2–2.5 min after the onset of milk let-down, as indicated by active infant swallowing. Mothers then manually expressed up to 20 ml of milk before resuming normal feeding. Mid-feed sampling was selected because it allows researchers to collect a smaller volume of milk, yet produces a sample with mean constituent concentrations that are not significantly different from a pooled, pumped content of an entire mammary gland [66–68]. Milk samples from Bolivia were collected over two research periods, and those collected in 2009 (*n* = 9) were collected using a different procedure. Mothers were asked to refrain from nursing from either breast for 1 h, instead of 2 hours, prior to a morning sampling. The sample breast was then fully evacuated with a manual breast pump [69]. Milk protein content is not significantly affected by time since last feed or sample expression mode, so differences in collection procedure for these samples are not expected to impact the results of this study [70].

All procedures were conducted with approval from respective institutional review boards and local advisory boards, where available. Ethical approval for all study procedures was granted by institutional review boards from Harvard University (Boston and Poland), University of Pennsylvania (Qom), University of California Santa Barbara (Tsimane), University of California Los Angeles (Himba), Northwestern University and University of San Carlos, Philippines (Cebu) and Washington University in St Louis and the Nepal Health Research Council (Nubri Tibetans). Details of all ethical approvals are provided in the Supplementary Material S1. Written or oral informed consent was obtained from all participants.

### Milk proteins analysis

Immune proteins were analyzed using triple-quadrupole time-of-flight mass spectrometry with an Agilent 6520 Q-TOF MS (Agilent Technologies, Inc., Santa Clara, CA) at the Lebrilla Lab at the University of California Davis. Whole milk samples were prepared and analyzed according to the protocol described by Huang *et al.* [71]. Immune protein concentrations are reported in mg/L.

### Statistical analysis

Two individuals were excluded from the dataset before statistical analysis. One individual from Argentina was excluded from analysis due to improbably low values of all immune proteins, likely due to a technical error during laboratory analysis. One individual from Bolivia/Tsimane was excluded due to extremely high levels of immune proteins consistent with mastitis, an exclusion criterion for participation. All summary statistics and results are presented on the analyzed sample (*n* = 164). All statistical analyses were performed in R version 3.1.0 [72]. Box-Cox power transformation was used to normalize protein concentrations before analysis, and transformed concentrations were examined graphically for normalization. Between-group principal components analyses were conducted to compare transformed protein concentrations among populations or subsistence patterns. Multivariate linear models were constructed using bgPCA response variables that cumulatively explained >90% of the variance. Infant sex, infant age and maternal parity were included in the models as confounding variables. Post-hoc pairwise comparisons modified from RVAideMemoire package using the ‘pairwise.manova’ function were used to assess differences in protein composition of milk in relation to population or subsistence pattern [73]. *P*-values were adjusted for multiple comparisons using the Holm method [74]. Effect sizes are reported as η^2^. Differences were considered statistically significant when *P* < 0.01. We employed a conservative threshold to be more cautious in our interpretations of the results [75].

## RESULTS

### Immune protein descriptives

**Figure 1.**
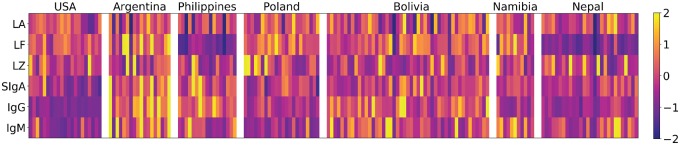
Milk immune protein concentrations visualized for all participants. This heatmap shows the relative protein concentrations for all individuals. Each column is an individual and each row is a protein (LA, lactalbumin; LF, lactoferrin; LZ, lysozyme; SIgA, Secretory Immunoglobulin A; IgG, Immunoglobulin G; IgM, Immunoglobulin M). Shading represents individual’s value as a z-score above (more yellow) or below (more purple) the mean for that protein across all individuals in the study

The concentrations of innate and acquired immune proteins varied across individuals (Fig. 1) and populations (Table 4), most notably among lactoferrin and the acquired immune proteins. Milk immune protein concentrations were correlated with one another. Innate immune proteins did not show a consistent pattern of correlation (Supplementary Material S2). Lysozyme was weakly negatively correlated with lactalbumin (*r* = −0.21, *P* = 0.005) and IgM (*r* = −0.19, *P* = 0.01). Lactalbumin was moderately positively correlated with lactoferrin (*r* = 0.34, *P* < 0.001). Lactoferrin and lactalbumin were also positively correlated with all of the acquired immune proteins (Lactoferrin-SIgA: *r* = 0.38, *P* < 0.001; Lactoferrin-IgG: *r* = 0.26, *P* < 0.001; Lactoferrin-IgM: *r* = 0.26, *P* < 0.001; Lactalbumin-SIgA: r = 0.2, *P* < 0.01; Lactalbumin-IgG: *r* = 0.34, *P* < 0.001; Lactalbumin-IgM: *r* = 0.24, *P* < 0.01). Acquired immune proteins were all positively correlated with each other (SIgA—IgG: *r* = 0.54, *P* < 0.001; SIgA–IgM: *r* = 0.44, *P* < 0.001, IgG—IgM: *r* = 0.34, *P* < 0.001).

**Table 4.** Summary of population immune protein concentrations

INNATE													
		Lactalbumin (mg/L)				Lysozyme (mg/L)				Lactoferrin (mg/L)			
*Country*	*n*	mean	SD	min	max	mean	SD	min	max	mean	SD	min	max
USA	21	570.39	64.49	436.84	667.65	18.13	11.07	5.36	54.77	436.63	87.53	258.04	613.78
Argentina	18	559.98	111.09	232.62	710.51	18.69	14.05	3.86	58.40	556.24	260.33	192.05	1082.19
Philippines	17	534.09	111.54	248.57	679.75	14.86	10.00	3.29	42.47	238.80	70.63	106.11	351.45
Poland	22	585.83	75.14	417.75	731.60	20.05	12.40	5.77	47.66	478.41	103.29	329.12	709.22
Bolivia	47	565.70	105.62	216.40	754.04	15.11	9.33	5.36	54.14	444.20	110.40	138.11	671.21
Namibia	11	574.83	67.81	484.73	702.72	16.51	9.23	3.67	35.00	464.73	163.45	194.06	731.16
Nepal	28	565.25	115.69	144.50	843.90	18.25	12.79	2.80	53.07	245.50	73.84	46.30	452.90
TOTAL	164	565.63	97.80	144.50	843.90	17.16	11.21	2.80	58.40	406.28	164.83	46.30	1082.19

ACQUIRED													
		SIgA (mg/L)				IgG (mg/L)				IgM (mg/L)			
*Country*	*n*	mean	SD	min	max	mean	SD	min	max	mean	SD	min	max
USA	21	79.18	41.28	23.50	199.49	6.27	2.49	3.36	12.93	4.23	3.73	0.37	15.29
Argentina	18	157.84	83.96	68.19	346.39	26.66	15.75	6.90	75.36	11.37	6.65	1.30	21.78
Philippines	17	145.70	112.24	60.67	534.60	20.97	8.83	10.64	48.42	7.62	4.88	1.61	20.16
Poland	22	95.47	39.36	14.05	164.49	11.66	4.16	6.18	20.65	4.38	3.18	0.79	14.27
Bolivia	47	108.16	39.08	39.37	242.97	18.99	9.20	4.73	46.59	9.05	6.11	1.99	32.44
Namibia	11	104.84	20.57	73.43	128.76	10.29	4.82	4.82	19.21	10.44	5.41	3.27	19.14
Nepal	28	99.84	31.81	28.51	163.68	9.97	4.01	1.69	18.34	8.28	6.49	1.01	33.85
TOTAL	164	110.45	59.68	14.05	534.60	15.30	10.24	1.69	75.36	7.87	5.90	0.37	33.85

### Populations

**Figure 2.**
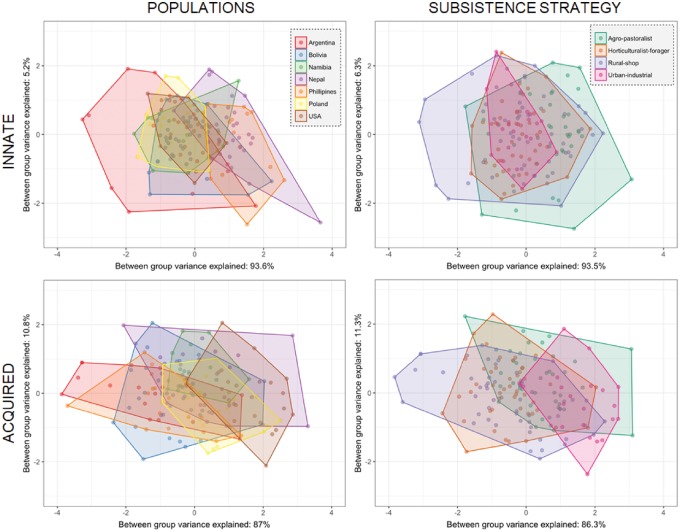
Between-group principal components analysis plots. The four plots display a visualization of the between-group principal components analysis used to create a composite measure of the composition of innate (top row) or acquired (bottom row) immune proteins for each population (left column) or subsistence group (right column). The shaded area depicts the convex hull for each population or subsistence group

The composition of innate immune proteins was less variable among populations than the composition of acquired immune proteins (Fig. 2). Innate immune protein composition was significantly different in 10 of 21 pair-wise comparisons between populations (Table 5). Comparisons that did not meet the threshold for statistically significant differences also had considerably smaller effect sizes (η^2^ ≤ 0.18 compared with η^2^ ≥ 0.41) and are therefore also less likely to represent biologically meaningful variation. Variation in the composition of innate immune proteins was due primarily to variance in lactoferrin concentration. Notably, average lactoferrin concentrations in the Filipino and ethnic Tibetan populations were approximately half the average concentrations of other populations in this study.

**Table 5.** Pair-wise comparisons of immune protein composition between populations: η^2^ values (*P*-values)

Country	USA	Argentina	Philippines	Poland	Bolivia	Namibia	Nepal
**USA**		0.12 (1)	**0.69 (<0.001)**	0.07 (1)	0.04 (1)	0.00 (1)	**0.62 (<0.001)**
**Argentina**	**0.57 (<0.001)**		**0.65 (<0.001)**	0.18 (0.411)	0.06 (1)	0.10 (1)	**0.51 (<0.001)**
**Philippines**	**0.64 (<0.001)**	0.10 (0.613)		**0.71 (<0.001)**	**0.57 (<0.001)**	**0.51 (0.005)**	0.02 (1)
**Poland**	**0.37 (0.002)**	**0.42 (<0.001)**	**0.35 (0.006)**		0.08 (0.852)	0.03 (1)	**0.63 (<0.001)**
**Bolivia**	**0.54 (<0.001)**	0.10 (0.193)	0.04 (0.613)	**0.29 (0.005)**		0.01 (1)	**0.53 (<0.001)**
**Namibia**	**0.46 (0.004)**	**0.51 (0.003)**	**0.57 (0.001)**	**0.38 (0.009)**	**0.23 (0.006)**		**0.41 (0.002)**
**Nepal**	**0.28 (0.006)**	**0.42 (<0.001)**	**0.41 (0.001)**	**0.29 (0.005)**	**0.23 (0.002)**	0.03 (0.613)	

The blue shaded boxes indicate the composition of innate immune proteins while the red shaded boxes indicate the composition of acquired immune proteins. Comparisons that are significantly different (*P* < 0.01) among subsistence patterns are bolded. *P*-values have been adjusted for multiple comparisons using the Holm method.

Acquired immune protein composition was significantly different in 17 of 21 pair-wise comparisons between populations (Table 5). Notably, samples from Boston (the only urban, industrialized population in this study) were significantly different from all other populations. Boston mothers have the lowest levels of all immunoglobulins (SIgA, IgG and IgM), with concentrations of IgG three to four times less than Qom, Tsimane or Cebu women and concentrations of IgM approximately two times less than the Qom, Tsimane, Nubri Tibetan and Himba women.

### Subsistence patterns

The composition of innate immune proteins was less variable among subsistence patterns than the composition of acquired immune proteins (Fig. 2). Innate immune protein composition significantly differed in two of six pairwise comparisons of subsistence patterns (Table 6). Pairwise comparisons that were significantly different were between horticulturalist-foragers and agro-pastoralists (η^2^ = 0.25 *P* < 0.001) and urban and agro-pastoralists (η^2^ = 0.25, *P* < 0.01). Acquired immune protein composition significantly differed in five of six pairwise comparisons of subsistence patterns (Table 6). The only non-significant difference was between horticulturalist-foragers and rural-shop (η^2^ = 0.04, *P* = 0.018).

**Table 6.** Pair-wise comparisons of immune protein composition between subsistence patterns: η^2^ values (*P*-values)

	Urban	Rural-shop	Horticulturalist-forager	Agro-pastoralist
**Urban**		0.01 (0.601**)**	0.13 (0.029**)**	**0.25 (0.002)**
**Rural-shop**	**0.37 (<0.001)**		0.09 (0.029)	0.10 (0.029)
**Horticulturalist-forager**	**0.56 (<0.001)**	0.04 (0.018)		**0.25 (<0.001)**
**Agro-pastoralist**	**0.33 (<0.001)**	**0.34 (<0.001)**	**0.26 (<0.001)**	

The comparisons in the blue shaded boxes are for the composition of innate immune proteins. The comparisons in the red shaded boxes are for the composition of acquired immune proteins. Comparisons that are significantly different (*P* < 0.01) among subsistence patterns are bolded. *P*-values have been adjusted for multiple comparisons using the Holm method.

## CONCLUSIONS AND IMPLICATIONS

Mother’s milk contains immune proteins that provide immunological protection and education for the developing infant. Examining milk from seven populations around the world, we found both innate and acquired immune protein composition in milk varied significantly among populations and subsistence patterns. Consistent with our predictions, differences between acquired immune protein compositions were more common than differences in innate immune protein composition among populations and subsistence patterns. Notably, population comparisons that were not different included populations with more similar geographic locations or subsistence strategies. These findings support the perspective that milk immunofactors vary among populations in part as a function of the pathogen pressures within that environment. Subsistence strategies influence pathogen exposure by engineering different local environments for pathogen growth, facilitating contact with varying pathogen sources and types, and/or providing different opportunities for exposure and transmission [28]. Immunofactors in human milk are therefore likely ‘tailored’ to the disease ecology that the infant is predicted to experience if pathogen exposure is consistent across generations. This would be consistent with theoretical models, which have demonstrated that for the maternal transfer of immunity to evolve; the risk of pathogen exposure of the mother and offspring must be correlated so that the mother’s immune defenses provide effective protection to the offspring [76]. These results motivate further research into how specific aspects of the maternal environment contribute to variation in milk immune proteins.

### Acquired immune proteins vary more than innate immune proteins

The composition of milk innate immune proteins showed fewer differences among populations and subsistence patterns than the composition of acquired immune proteins. Acquired immune proteins significantly differed in 17 of 21 pairwise comparisons between populations, whereas innate immune proteins were significantly different in only 10 of 21 pairwise comparisons. This is consistent with what we would expect based on how the different branches of the immune system have evolved to defend against pathogens. Acquired immune proteins are produced by an individual in response to specific pathogen exposures, whereas innate immune responses are encoded in the genome, utilizing pattern recognition receptors that are often structurally and functionally conserved across vertebrates and invertebrates [77]. Thus, the acquired immune molecules in a mother’s milk reflect the pathogens she has encountered and that are likely to endanger her infant [78]. Due to immunological memory, antibodies that are present in human milk can reflect not only a woman’s recent exposures, but also exposures from across her lifetime. The specific antibodies present in her milk; however, will depend on the type of pathogens she has encountered and the timing of those encounters. Antibody isotypes (i.e. IgA, IgG and IgM) have specialized functions and different antibody isotypes are produced in response to different types and locations of pathogens [79]. The longitudinal maintenance of antibody responses also varies among specific pathogens [80]. Immigrant mothers in the UK had much higher levels of milk antibodies specific to strains of *Escherichia coli* common on the Indian subcontinent than non-immigrant white mothers, and antibody level was not correlated with time since immigration [20]. In contrast, Novak and Svennerholm found Bangladeshi women had higher, less seasonably variable levels of rotavirus-specific milk SIgA than did Swedish women [81]. The authors posited that this difference was observed because while there is a single annual peak in rotavirus infections in Sweden, there are two annual peaks in Bangladesh. More frequent exposures to rotavirus likely maintain higher and more stable concentrations of rotavirus-specific antibodies in the Bangladeshi mothers’ milk [81].

The pathogen pressure encountered within the local environment depends on geography, culture, and the availability of sanitation and western medical services. In this study, between-population comparisons that were not different included populations that shared aspects of their cultural ecology. For example, the Himba of Namibia and ethnic Tibetans in the Nubri Valley of Nepal are geographically distant but both practice agro-pastoralism. Populations that share a subsistence pattern may be more likely to face similar pathogens and commensal microbes as a result of shared exposures (e.g. domesticated animals, population density) or behaviors (e.g. soil cultivation, consumption of raw milk). In a recent study, Obregon-Tito and colleagues found that populations that shared a subsistence strategy (hunter-gatherer, traditional agriculturalist or urban-industrialist) had similar gut microbial profiles despite being on different continents [38]. Thus, comparing the composition of milk across subsistence patterns rather than countries or populations may be a more bioculturally meaningful comparison. The acquired immune protein composition of milk differed significantly between all subsistence patterns except rural-shop and horticulturalist-foragers.

In this study, the only group representing horticulturalist-foragers, the Tsimane of Bolivia, reside in a neighboring country to one group of the rural-shop (Qom of Argentina). Innate and acquired immune composition also did not significantly differ between the Tsimane and Qom populations. This is consistent with results from a recent study that found that the presence and concentration of immunofactors in milk varied with geographic location [45]. As populations located in closer geographic proximity tend to share more recent common ancestry, populations on the same continent tend to be more genetically similar to each other than to geographically distant populations [82]. Moreover, Native Americans have lower genetic diversity than indigenous populations on other continents [83]. Thus, the Qom and Tsimane are expected to be the most closely related populations included in this study and it is possible this might contribute to similarity in their milk composition. The concentrations of some milk constituents, such as human milk oligosaccharides, are influenced by maternal genotype [84]. Variability in adult immune function, however, is generally influenced more by environmental than heritable factors, and this is particularly true for the acquired immune system [85, 86]. Ruiz *et al.* [45] found that some milk immune factors were significantly different between rural and urban populations in Gambia. This suggests that the composition of milk immunofactors among populations may be more influenced by maternal environment than genetic relatedness [45].

Though not directly considered here, geography is also an important influence of pathogen species and diversity [23]. Geographic proximity, however, is unlikely to fully explain the similarity in milk composition between these populations as they reside in different biomes with overlapping, but distinct, endemic pathogens [87]. The Tsimane live in the tropical lowlands while the Qom live in Gran Chaco region, which is characterized by dry shrubland vegetation, savannah grasslands, and semi-arid forests [58, 88, 89]. Although respiratory and gastrointestinal infections are common among both the Qom and Tsimane, populations living in tropical forests are particularly susceptible to helminth infections and Tsimane often have multiple, co-occurring parasitic infections [58, 88, 90].

Alternatively, similarities in milk composition among these populations may be explained by similarities in their household environments. Household features such as flooring type or water source affect pathogen exposures and health outcomes [40]. Though household features are not directly linked to specific subsistence strategies, traditionally living populations tend to retain their traditional home structures and have less sanitary infrastructure such as running water or indoor toilets than industrialized populations or populations with mixed subsistence economies. Though the Qom mothers sampled reside in a peri-urban barrio and no longer practice traditional subsistence strategies, many still have dirt floors and lack indoor toilets, similar to Tsimane households [5]. However, these features are also shared with the Himba, who had different acquired immune protein composition in their milk than the Tsimane and Qom. This suggests that mode of subsistence leaves a signature in milk despite other shared features of the mothers’ local environments. Studies of the built environment have focused on the effects of physical structures on health, but household microbial communities are also influenced by human activity [91, 92]. Previous studies have found that farmers carry fungal spores from cow barns into their homes, and the interactions of agro-pastoralists with cattle may contribute to diverse household microbial environments from other modes of subsistence in similar ways [93].

Differences in acquired immune protein composition in this study were primarily explained by variation in IgG. There was a more than threefold difference in mean IgG concentrations among the populations studied. IgG antibodies predominate in blood and extracellular fluid, where they protect against viruses, bacteria, and toxins [79]. IgG is present at low concentrations in human milk and is thought to be less important than SIgA for passive immune defense in human milk as much larger quantities of maternal IgG are transferred across the placenta during late pregnancy [94]. Due to its abundance in human milk and vital role in mucosal immune protection, SIgA is often the focus of human milk studies. Higher concentrations of SIgA tend to be found in women with a higher current microbial load or lower socioeconomic status [9, 42, 95, 96, but see 45]. SIgA and IgG may also increase in response to active infection in the mother or infant [97, 98, but see 5]. In this study, US women had the lowest levels of all acquired immune proteins measured. The US also has the lowest child mortality rates and percentage of child deaths due to infectious causes of the populations studied [99, 100]. Mothers from countries with high levels of child mortality (Namibia, Bolivia and Nepal) or higher percentages of child deaths due to infection (Bolivia, Nepal and Philippines) tended to have higher average concentrations of acquired immune proteins. Assuming that infant mortality rates correlate with pathogen load, our results are consistent with previous research that milk SIgA concentrations are positively associated with environmental pathogen load [9, 42, 95, 96]. Importantly, country-wide estimates of child mortality are likely underestimates for the Qom, Tsimane, Nubri Tibetans and Himba. Indigenous populations worldwide have poorer health, including more infectious diseases, than non-indigenous persons, even in high-income nations [101].

### Sources of variation in milk innate immune proteins

Although acquired immune protein composition was more variable among populations, innate immune proteins also varied to some extent among both populations and subsistence patterns. Innate immune proteins were significantly different in 10 of 21 pairwise comparisons among populations, and agro-pastoralists had significantly different milk innate immune protein composition than urban-industrialists and horticulturalist-foragers. Though innate immune defenses are expected to be more conserved than acquired immune defenses, innate immune function is also variable across populations. In part, differences in immune function among populations may have a genetic basis. Locally prevalent pathogens have been an important selective force, especially on immunity-related genes, as humans have migrated across a diverse range of landscapes and developed new subsistence strategies [102, 103]. Immune function is also variable among individuals and populations due to evolved reaction norms that allow individual immune responses to vary among environmental contexts (reviewed in [85]). Among the environmental interactions that shape individuals’ immune phenotypes are interactions between the immune system and local pathogens. For example, gram-negative bacteria tend to activate innate immune responses, while viral infections tend to be more effectively suppressed by acquired immune responses (reviewed in [104]). Pathogens typically elicit Th1 or Th2 type immune responses. As Th1 and Th2 immune responses inhibit each other, repeated exposure to pathogens that elicit one type of response can lead to polarization of the immune system and constrain an individual’s ability to effectively mount immune responses to co-occurring infections (reviewed in [85]). Parasites have also been shown to modulate the immune systems of their hosts [105, 106]. Notably, helminths have been found to suppress several types of immunological responses in ways that not only permit tolerance of the parasite, but can also reduce vaccine responses and the ability to resist other infections [105]. As the effects of helminth infections on host immunity may remain even in the absence of active helminth infections, helminths may be an important modulator of immune function in communities where these infections are common [106]. Differential immune function among populations may also be understood through an evolutionary life history framework [18]. According to this framework, pathogen prevalence, nutritional availability, and extrinsic mortality cues inform trade-offs in investment between the innate and acquired immunity during sensitive periods in immune development which may calibrate ‘set points’ for immune function in later life [18]. It is not yet fully understood, however, if or how factors that influence systemic innate immune function are reflected in milk composition.

Much of the variance in innate immune protein profiles in this study is attributable to lactoferrin. Lactoferrin comprises 15–20% of the total protein in human milk and has several immunological functions, including bacteriostatic, bactericidal and anti-inflammatory activities [11]. A recent review found lactoferrin varied among geographic locations; however, in contrast to our study, lactoferrin concentrations were higher in Asia than other parts of the world [107]. Lactoferrin concentrations in Nubri Tibetan and Cebu mothers’ milk are low compared with other populations in this study, but within the range of average concentrations of lactoferrin reported in mature human milk (2–4 g/L, reviewed in [108]). Lactoferrin concentrations generally decrease during the first month of lactation and then are stable across lactation (reviewed in [107]), but infant age was consistent across populations and statistically controlled for in our models, so it cannot account for the differences reported here. Other factors that influence lactoferrin concentrations are still not fully understood. Parity has been positively [109] and negatively [110] correlated with milk lactoferrin concentrations, though our results are consistent with more recent work that found no relationship [111]. Maternal dietary quality may impact lactoferrin, though the direction of this relationship is not clear. Higher levels of lactoferrin may be related to a diet high in iron [112]. Anemic or poorly nourished women have been found to have milk lactoferrin concentrations lower [109, 113], higher [114] or similar [111, 115] as among non-anemic or well-nourished women. Higher lactoferrin levels have also been associated with maternal or infant illness [5, 97, but see 98]. These studies suggest lactoferrin concentrations are affected by maternal environment, but further research is needed to fully understand how lactoferrin reflects disease and nutritional ecology.

### Strengths and limitations

This study is among the first investigations of milk immunofactors among diverse populations. A notable strength of this study is that all milk samples were analyzed at the same time in the same laboratory. This reduces measurement error and allows us to make direct comparisons among populations. Interassay and interlaboratory differences can make it difficult to determine the extent of biological variation among groups, but comparable results are needed to develop effective clinical and public health guidelines [116, 117].

Although comparisons of milk immunofactor concentrations can inform us about the variation in milk across populations, our study has limitations. A significant limitation in this study is that it is difficult to interpret the causes of variation in total antibody concentrations. For example, while the Qom had the highest average concentration of all milk acquired immune proteins, estimated infant mortality rates in the Qom (18.6 per 1000 live births) are lower than the Himba, Tsimane, and Nubri Tibetans [54, 58, 118, 119]. One possible interpretation is that the Qom have greater exposure to pathogens than Himba, Tsimane or Nubri Tibetans, but this would seem to contradict what we know about these populations’ environments (Table 2). Alternatively, higher antibody concentrations could indicate that the Qom produce larger antibody responses to the pathogens they do encounter. High antibody concentrations could therefore indicate that those individuals or populations are able to allocate more energy to mounting immune responses (reviewed in [120, 121]). The data available here are not able to conclusively differentiate the extent to which we may be measuring greater antibody responses to similar exposures or similar antibody responses to different exposures. These results motivate further research to understand how maternal nutritional and disease ecology interact to shape the composition of milk immune proteins.

This study is also limited by small, uneven sample sizes that were collected for different primary research aims. As a result, we cannot consistently control for individual health histories or heterogeneity in living conditions within populations. We also cannot control for milk volume due to logistical difficulties collecting accurate volume measurements in field settings. Milk volume can vary considerably among individuals and may vary more than milk composition in response to changes in maternal condition (reviewed in [122]). Both milk volume and concentrations are needed to determine the total transfer of milk components to the child, arguably the most biologically relevant measure [67]. However, indirect test weighing over 24 h (the gold standard for assessing milk volume) can be extremely challenging or impractical in field settings [55, 67]. Finally, this study examines only a few of the numerous milk immunofactors that have been identified to date. Immune proteins are a small subset of the immunofactors in human milk, and among the best-studied within the understudied domain of milk [123–125]. As many immune defenses have functional redundancy, differences in selected immunofactor concentrations may not translate to differences in the quality of immune protection afforded to the infant by mother’s milk.

### Implications and future directions

Our results show that the composition of innate and acquired immune proteins in human milk differs among populations and by mode of subsistence. These results add to the existing literature investigating variation in milk composition among mothers living in different physical and cultural environments and, to our knowledge, this is the first study to examine how milk protein composition varies among subsistence strategies. Subsistence strategies have influenced diet and pathogen exposure throughout human evolutionary history and are thus likely to have shaped the composition of mothers’ milk. Of particular importance for designing public health interventions, we found further evidence that mother’s milk from western, urban-industrial populations differs from women in non-industrialized populations, including populations undergoing economic transitions (in this study, those categorized as ‘rural-shop’) [45, 126, 127]. Very few populations still exclusively practice traditional subsistence strategies, and populations are likely to face health challenges as their diets, environments and physical activity patterns change with increasing market integration [128–130]. Future investigations showing the extent to which milk composition responds to these changes may help us understand the early life origins of ‘mismatch’ diseases, particularly those involving the immune system.

Further study is needed to understand what specific aspects of the local environment influence the composition of milk immune proteins. In this study, agro-pastoralists had different acquired and milk innate immune protein composition than urban-industrialists and horticulturalist-foragers. Exposure to livestock (particularly ungulates), their products (milk and meat), their waste, and associated pathogens in these populations may contribute to the variation in immune protein composition observed among subsistence patterns, as neither the horticulturalist-foragers nor urban-industrialists have regular contact with large livestock. In traditionally living pastoralist communities, contact with livestock generally increases rates of parasitic infections (reviewed in [131]). Traditionally living pastoralist communities often also have other features of their local environments that make it difficult to disentangle the effects of domesticated animal exposure on milk composition from other exposures, such as reduced access to clean water or medical care. Comparing groups with differing subsistence strategies but similar exposures, e.g. pastoralists and traditional agriculturalists who both have regular contact with livestock may allow us to better identify how specific aspects of the maternal environment relate to milk composition.

Research is also needed to understand the effects of variation in human milk composition on infant health outcomes. Bioactive molecules passed through milk provide protection against infection while the infant’s own immune defenses are becoming competent. These molecules also signal the infant’s immune system during a critical period in early life that may calibrate immune responses throughout life [132, 133]. Understanding how variation in the composition of human milk impacts infant health outcomes may allow us to better understand the biological significance of the differences in milk composition we observe in this study and may also help elucidate the early life origins of variation in adult immune function across populations.

## FUNDING

This research was funded with a Harvard GSAS Graduate Society Summer Fellowship to L.K., a National Science Foundation (NSF) Doctoral Dissertation Improvement Grant (BCS-1232370) and a Wenner-Gren Foundation Dissertation Fieldwork Grant to M.M. The Tsimane Health and Life History Project is supported by funding from the National Institute of Health/National Institute of Aging (NIH/NIA R01AG024119-01) to M.G. and H.K. Cebu research was funded by a NSF Doctoral Dissertation Improvement Grant (BCS-0746320) to E.Q.; Nubri, Nepal research by the Leakey Foundation and Wenner-Gren Foundation awards to E.Q.

## Supplementary Material

Supplement 1

Supplement 2
