# Supplementary material for: Variation among populations in the immune protein composition of mother’s milk reflects subsistence pattern
Source: Evol Med Public Health. 2018 Oct 13;2018(1):230–45. doi: 10.1093/emph/eoy031 (PMC6222208; doi:10.1093/emph/eoy031)
Supplement: Supplement 1 [file eoy031_supplement_1.pdf]

S1. SUPPLEMENTAL POPULATION INFORMATION

URBAN – INDUSTRIAL

USA: US samples were collected from women living in the Greater Boston area in summer 2013. These women represent a W.E.I.R.D. (Western, Educated, Industrialized, Rich, Democratic) population [134]. According to the 2010 US Census, Boston-Cambridge-Newton was the 10th largest metropolitan statistical area in the US, with a population of around 4.5 million [135]. Nearly all women who participated in this study identified as white, and all had completed a college degree. Many participants also reported at least some post-secondary education. In 2013, the population of this area was approximately 73% White, 10% Hispanic, 7% Black, and 7% Asian [136]. Thus, this sample group is not reflective of US as a whole, but is likely to be reflective of women who breastfeed in the communities around Harvard University.

Ethical approval for work in Boston was granted by the Harvard University Committee on the Use of Human Subjects (#23868).

RURAL - SHOP

ARGENTINA: Argentinian samples were collected from indigenous Qom (also called Toba in previous literature) women in northeastern Argentina from September 2012 to March 2013. Traditionally, the Qom people were hunter-gatherers, but today many have migrated to poor peri-urban barrios [48]. Participants were recruited in Namqom, a village located 11 km NW of the city of Formosa, which has a population of approximately 3000 people distributed in an area of 150 hectares [5]. Approximately half of the homes in the community have dirt floors, and no households have indoor toilets. Drinking water is available from shared taps and most cooking takes place outside over open fires. While peri-urban and urban Qom families may hunt, fish, or

gather opportunistically, most households primarily depend on wages from men's labor or government subsidies [58]. Women's activities typically include household chores, children caretaking, and basket weaving. Some women also participate in the market economy, either through employment such as cooks and teachers' assistants or by selling handicrafts [137].

The provincial government offers free healthcare at local clinics, including pre- and postnatal care programs. Nearly all infants are born at hospitals, however infant and child mortality rates are high and largely due to preventable causes [58]. Infant illnesses, such as respiratory and diarrheal infections, have previously been reported to be common among this group of participants [5]. Tuberculosis, urinary infections, and gastrointestinal infectious are common among adults in this community [58]. Obesity rates among Qom adults are also increasing as a result of dietary and lifestyle changes, and nearly half of adult women are overweight or obese [138].

Ethical approval for work with the Qom was granted by the University of Pennsylvania Institutional Review Board (#811200).

PHILIPPINES: Filipino samples were collected in collaboration with the Cebu Longitudinal Health and Nutrition Survey (CLHNS) during September 2007 to March 2008. The CLHNS is an ongoing birth cohort study of individuals in 33 communities within the Cebu metropolitan area [139, 140]. Metropolitan Cebu is the second-largest metropolitan area in the Philippines, with a total population of around 2 million [140]. Half of the participants involved in this study were recruited from urban areas where residents primarily engaged in wage labor, and half from surrounding agrarian communities that practiced small-scale farming. As there was variation in the urbanicity of this sample, households varied somewhat among urban and rural communities.

While concrete or bamboo floors are common in both urban and rural areas, indoor plumbing is not found in rural homes but is sometimes present in urban homes. Participants also varied in their access to small neighborhood convenience stores and larger grocery stores [141]. However, both groups had access to a variety of commercially produced foods as well as local produce, eggs, and fish. It is not uncommon for women to own or work in small neighborhood convenience stores, which often have many snack foods [142].

As in many developing countries, increasing household incomes and ownership of consumer goods in this population have been associated with increasing rates of overweight and obesity [142]. However, infectious diseases continue to be a leading cause of mortality in Southeast Asia, including the Philippines [143]. Respiratory infections are the third leading cause of death for both adults and children under five in the Philippines [144].

Ethical approval for work with the Cebu was granted by Northwestern University and the University of San Carlos, Philippines (both #STU00001299).

POLAND: Polish samples were collected from women living in the area of the Mogielica Human Ecology Study Site in summer 2012. The Mogielica Human Ecology Study Site encompasses a group of rural villages in Beskid Wyspowy mountain range of southern Poland [145]. Households in this region have historically engaged in small-scale agriculture, but are increasingly and rapidly transitioning to participation in wage labor [51]. Homes in this region are typically modern, with indoor plumbing and modern heating systems. Though less than 5% of households in the area exclusively subsisted from farming by 2009-2010, most households still participate in agricultural activities to some degree [51]. Commonly grown crops include wheat, rye, potatoes, vegetables, and fruits such as raspberries and plums [146]. When

conducted, women participate in the highly seasonal and labor-intensive agricultural work [50]. Most households also keep a cow or chickens [146]. Food that is not grown or produced by the households is typically purchased from small local shops.

Nearly all Polish citizens have compulsory health insurance through government programs [147]. Modern medical care at the Mogielica Human Ecology Study Site is available at several local clinics and a small hospital that is accessible by local bus routes. Since 1989, country-level health indicators such as life expectancy and infant mortality rates have improved but are still lower and higher, respectively, than many other European countries [148]. Chronic conditions such as cardiovascular disease, respiratory diseases, and cancers among the most common causes of death in Poland [147].

Ethical approval for work in Poland was granted by the Harvard University Committee on the Use of Human Subjects (#21979, #IRB13-0900).

HORTICULTURALIST- FORAGERS

BOLIVIA: Bolivian samples were collected from indigenous Tsimane women during 2009 and 2012. The Tsimane are a high-fertility forager-horticulturalist population (~15,000) residing in the Bolivian lowlands (southwestern Amazon Basin). The Tsimane live in open thatched-roof housing structures consisting of individual family and extended kin clusters; village sizes range from 30-500 individuals [61]. In addition to hunting and fishing, the Tsimane grow plantains, rice, corn/maize, and manioc/cassava using slash-and-burn horticulture [36]. They also have small livestock, such as pigs and chickens [148]. Most Tsimane obtain water from rivers and streams, and rarely boil water before drinking it. The average adult diet is comprised of 74%

plant and 26% animal foods, with market foods (pasta, bread, beef jerky, etc.) comprising < 4% of total dietary energy [69].

The Tsimane have minimal access to regular health care or medications [149]. Hygienic conditions are generally poor and helminthic, protozoal, fungal, and other infections are common throughout life [150]. The average life expectancy is 54 years, and approximately half of all deaths since 1950 are due to infectious diseases [36, 149]. Stunting rates are high, likely due to poor dietary quality and high infectious disease burden, but Tsimane are generally are not malnourished [148].

Ethical approval work with the Tsimane was granted by the UC-Santa Barbara Human Subjects Committee (ANTH-GU-MI-010-3U #09-312, ANTH-GU-MI-010-10R #10-290, ANTH-GU-MI-010 #19-13-0206, ANTH-GU-MI-010 #3-15-067).

AGRO-PASTORALISTS

NAMIBIA: Namibian samples were collected from indigenous Himba women during September 2013. The Himba people are semi-nomadic agro-pastoralists who reside in the arid, northwest corner of Namibia in a part of the Kunene region referred to as the Kaokoveld [52]. There are an estimated 20,000 Himba currently living in Kaokoland. The fieldsite for this project is in the Omuhonga Basin, about 150 km from the main district town of Opuwo. The Himba live in extended family households. Polygyny is common and co-wives maintain separate huts within the same household and co-wives often share productive and reproductive labor [151]. Out of wedlock births and infidelity are also frequent [52]. The Himba primarily depend on livestock for both subsistence and trade, and both men and women participate in the care of cattle, goats, and sheep [52]. Some foraging still occurs, particularly during drought years, mainly for palm

nuts and honey. Women have also recently begun growing maize and millet in seasonal gardens [118, 152].

Access to modern healthcare is limited. There is one small clinic a day's walk from the study area [118]. However, routine medical care is not common and many pastoralists in the area do not know their exact age [153]. Most women continue to give birth at home with the assistance of kin or midwives [118]. Contraceptive knowledge is scarce, so this is still considered a "natural fertility" population [52]. Similar to other pastoralist populations, infant mortality rates have been estimated to be high, between 12 and 48% of all births [118]. The Himba have high levels of STI infections, and infections are more common in women than men [153].

Ethical approval for work with the Himba was granted by the University of California Los Angeles IRB (IRB#13-000881).

NEPAL: Nepalese samples were collected from ethnic Tibetans living in Nubri Valley during the summer of 2013. The Nubri Valley is located in the Gorkha District of Nepal and is part of the Himalayan mountain range [55]. Participants were recruited from rural villages at high altitude, between 2,090-3,830 m [154]. The Nubri Tibetans are agro-pastoralists who herd yaks and yak-cow crossbreeds and grow crops including barley, potatoes, and maize [54, 154]. Most residents of the area live in two-story homes in which the animals live on the ground floor and the family lives in the second story. In addition to traditional subsistence activities, Nubri residents also trade timber and medicinal plants and participate in a developing tourism industry [54, 154, 155]. Educational and health services in this area are largely undeveloped [55, 156].

Ethical approval for work with the Nubri was granted by Washington University in St. Louis Institutional Review Board (#201302059) and the Nepal Health Research Council (#1329).

References

134. Henrich J, Heine SJ, Norenzayan A. 2010. Most people are not WEIRD. *Nature* 2010;**466**:29.refe
135. US Census Bureau, Population Division. *Annual estimates of the resident population: April 1, 2010 to July 1, 2011.* https://factfinder.census.gov/faces/tableservices/jsf/pages/productview.xhtml?src=bkmk (3 January 2018, date last accessed).
136. Data USA. *Boston-Cambridge-Newton, MA-NH Metro Area.* https://datausa.io/profile/geo/boston-cambridge-quincy-ma-nh-metro-area/#economy (3 January 2018, date last accessed).
137. Valeggia C. Changing times for the Argetine Toba: who cares for the baby now? In: Bentley G, Mace R (eds.) *Substitute Parents: Biological and Social Perspectives on Alloparenting in Human Societies*. New York: Berhahn Books, 2009, 100-14.
138. Valeggia C, Ellison PT. Nutrition, breastfeeding, and fertility: Changing lifestyles and policy implications. *DRCLAS News,* Harvard University, 2001, 24-7.
139. Quinn E, Largado F, Borja J *et al.* Maternal Characteristics Associated with Milk Leptin Content in a Sample of Filipino Women and Associations with Infant Weight for Age. *J Hum Lact* 2015;**31**:273–81.
140. Adair L, Popkin BM, Akin JS, *et al.* Cohort profile: The Cebu Longitudinal Health and Nutrition Survey. *Int J Epidemiol* 2011;**40**:619-625.
141. Dahly DL, Adair L. Quantifying the urban environment: a scale measure of urbanicity outperforms the urban-rural dichotomy. *Sco Sci Med* 2007;**64**(7):1407-1419.
142. Adair L. Dramatic rise in overweight and obesity in adult Filipino women and risk of hypertension. *Obesity* 2004;**12**(8):1335-1341.
143. McDade T, Rutherford J, Adair L *et al.* Population differences in associations between C-reactive protein concentration and adiposity: comparison of young adults in the Philippines and the United States. *Am J Clin Nutrition* 2009;**89**:1237–45.
144. World Health Organization. *Philippines: WHO statistical profile*. http://www.who.int/gho/countries/phl.pdf?ua=1 (4 January 2018, date last accessed).
145. Jasienska G. The Fragile Wisdom: An Evolutionary View on Women's Biology and Health. Cambridge: Harvard University Press, 2013.
146. Colleran H. Farming in transition: land and property inheritance in a rural Polish population. *Soc Biol Hum Aff* 2014;**78**:7-19.
147. Sagan A, Panteli D, Borkowski W, *et al*. Poland: Health system review. *Health Sys Transit* 2011, **13**(8):1–193.
148. Foster Z, Byron E, Reyes-Garcia V *et al.* Physical growth and nutritional status of Tsimane Amerindian children of lowland Bolivia. Am J Phys Anthropol 2005;**126**:343-51.
149. Gurven M, Kapaln H, Winling J, *et al.* Mortality experience of Tsimane Amerindians of Bolivia: regional variation and temporal trends. Am J Hum Biol 2007;**19**:376-98.

150. Martin M, Blackwell AD, Gurven M. Make new friends and keep the old? Parasite coinfection and comorbidity in *Homo sapiens*. In: Brinkworth JF, Pechenkina K (eds). *Primates, Pathogens, and Evolution.* New York: Springer Science, 2013, 363-87.
151. Scelza BA. Perceptions of Polygyny: The Effects of Offspring and Other Kin on Co-Wife Satisfaction. *Biodemography Soc Biol* 2015;**61**(1):98-110.
152. Scelza B, Prall S. Partner preferences in the context of concurrency: What Himba want in formal and informal partners. *Evol Hum Behav* 2017;**39**(2):212-19.
153. Hazel A, Foxman B, Low B. Herpes simplex virus type 2 among mobile pastoralists in northwestern Namibia. *Ann Hum Biol* 2014;**42**:543–51.
154. Quinn E, Childs G. Ecological pressures and milk metabolic hormones of ethnic Tibetans living at different altitudes. *Ann Hum Biol* 2016;**44**:1–12.
155. Childs G, Choedup N. Indigenous management strategies and socioeconomic impacts of Yartsa Gunbu (*Ophiocordyceps sinensis*) harvesting in Nubri and Tsum, Nepal. *Himalaya* 2014;**34(**1):7.
156. Childs G, Craig S, Beall CM, *et al.* 2014. Depopulating the Himalayan highlands: education and outmigration from ethnically Tibetan communities of Nepal. *Mt Res Dev* 2014;**34**(2):85-94.
