# Supplementary material for: Variation among populations in the immune protein composition of mother’s milk reflects subsistence pattern
Source: Evol Med Public Health. 2018 Oct 13;2018(1):230–45. doi: 10.1093/emph/eoy031 (PMC6222208; doi:10.1093/emph/eoy031)
Supplement: Supplement 2 [file eoy031_supplement_2.docx]

**Table S1. Correlations of milk immune protein concentrations.** The table below shows the Pearson correlation coefficients for between pairs of milk immune protein concentrations. Blue shading covers correlations among innate immune proteins, red shading covers correlations among adaptive immune proteins, and purple shading covers correlations between adaptive and innate immune proteins.

|  | ***Lysozyme*** | ***Lactoferrin*** | ***Lactalbumin*** | ***SIgA*** | ***IgG*** | ***IgM*** |
| --- | --- | --- | --- | --- | --- | --- |
| ***Lysozyme*** | 1 |  |  |  |  |  |
| ***Lactoferrin*** | 0.05 | 1 |  |  |  |  |
| ***Lactalbumin*** | -0.21** | 0.33*** | 1 |  |  |  |
| ***SIgA*** | 0.02 | 0.38*** | 0.2** | 1 |  |  |
| ***IgG*** | -0.08 | 0.26*** | 0.34*** | 0.54*** | 1 |  |
| ***IgM*** | -0.19** | 0.26*** | 0.24** | 0.44*** | 0.34*** | 1 |

Statistical Significance: * = p<0.05, ** =p≤0.01, *** = p≤0.001
